# An early regulatory mechanism of hyperinflammation by restricting monocyte contribution

**DOI:** 10.3389/fimmu.2024.1398153

**Published:** 2024-07-08

**Authors:** Megumi Akiyama, Masashi Kanayama, Yoshihiro Umezawa, Toshikage Nagao, Yuta Izumi, Masahide Yamamoto, Toshiaki Ohteki

**Affiliations:** ^1^ Department of Biodefense Research, Medical Research Institute, Tokyo Medical and Dental University (TMDU), Tokyo, Japan; ^2^ Department of Hematology, Graduate School of Medical and Dental Sciences, TMDU, Tokyo, Japan

**Keywords:** monocyte, apoptosis, inflammation, cytokine, CRS - cytokine release syndrome

## Abstract

Innate immune cells play a key role in inflammation as a source of pro-inflammatory cytokines. However, it remains unclear how innate immunity-mediated inflammation is fine-tuned to minimize tissue damage and assure the host’s survival at the early phase of systemic inflammation. The results of this study with mouse models demonstrate that the supply of monocytes is restricted depending on the magnitude of inflammation. During the acute phase of severe inflammation, monocytes, but not neutrophils, were substantially reduced by apoptosis and the remaining monocytes were dysfunctional in the bone marrow. Monocyte-specific ablation of *Casp3/7* prevented monocyte apoptosis but promoted monocyte necrosis in the bone marrow, leading to elevated levels of pro-inflammatory cytokines and the increased mortality of mice during systemic inflammation. Importantly, the limitation of monocyte supply was dependent on pro-inflammatory cytokines *in vivo*. Consistently, a reduction of monocytes was observed in the peripheral blood during cytokine-release syndrome (CRS) patients, a pathogen-unrelated systemic inflammation induced by chimeric antigen receptor-T cell (CAR-T cell) therapy. Thus, monocytes act as a safety valve to alleviate tissue damage caused by inflammation and ensure host survival, which may be responsible for a primitive immune-control mechanism that does not require intervention by acquired immunity.

## 1 Introduction

Inflammation is a basic biological phenomenon that is mediated by the immune system and therefore has been studied for a long time. Although inflammatory responses are necessary to maintain homeostasis (1), they can be a serious risk to life and health if inappropriately driven and sustained. Before the COVID-19 pandemic, about 19.7% of deaths were related to sepsis, a life-threatening multiple organ failure induced by infection-mediated inflammation (2), in the world (3). After the pandemic, the mortality of COVID-19 patients with severe sepsis reached up to 48% (4). In addition to infection-induced inflammation, immune therapies such as chimeric antigen receptor-T cell (CAR-T cell) treatment of patients with hematological malignancies often cause an acute systemic inflammation known as cytokine release syndrome (CRS), which sometimes threatens the lives of patients. Thus, regardless of pathogen involvement, controlling severe systemic inflammation remains the biggest challenge even today.

Innate immunity plays a critical role in host protection by phagocytosing pathogens, producing cytokines and inducing adaptive immunity. However, at the same time, the production of systemic cytokines by activated innate immune cells causes tissue damage and the dysfunction of multiple organs during systemic infection, which could be a life-threatening risk (5–7). Especially in the early stage of inflammation, the mechanism of innate immunity to regulate the balance between host protection and inflammation-mediated tissue damage has not been fully understood.

Monocytes are a unique immune cell population that can differentiate into multiple cell lineages such as macrophages and dendritic cells (8), even though they are constantly provided to the periphery through circulating blood (9). Classical monocytes, a major subset of monocytes, are related to the pathogenesis of various diseases such as cancer, infection and inflammation by producing cytokines and differentiating into pro-inflammatory and into anti-inflammatory cell populations (10–15). In the early stage of infection, monocytes are recruited from the bone marrow (BM) to infected sites and play roles in inflammation and pathogen clearance (10, 16). Indeed, genetic deletion of *Ccr2*, a gene encoding a critical chemokine receptor for monocyte exit from the BM to the periphery, decreased the severity of sepsis after cecal ligation and puncture (CLP) induction with reduced systemic cytokine production, suggesting that existing monocytes in the periphery are important for the progression of inflammation (17). In addition, the tolerance and trained memory of monocytes modify the secondary immune responses at the late phase of inflammatory responses including microbial infections (18). However, the early regulatory mechanism of excessive inflammation that affects the fate of monocytes has remained unclear.

In this study, we found that the fate of monocytes is dramatically changed to maintain homeostasis depending on the intensity of systemic inflammation. At early stages of severe systemic inflammation, the number of monocytes was significantly reduced in peripheral organs although neutrophils were successfully supplied to the periphery. This was because half of the monocytes were immediately killed by apoptosis, and the remaining monocytes were dysfunctional and failed to migrate into the periphery. Importantly, the mechanism to prevent monocyte supply was pro-inflammatory cytokine-dependent and driven only when severe inflammation was induced. The monocyte-specific ablation of *Casp3* and *Casp7* prevented their apoptosis but promoted monocyte necrosis, which increased both the production of pro-inflammatory cytokines and the mortality of mice during sepsis. Finally, we collected blood samples of CRS patients who received CAR-T cell therapy and confirmed that the classical monocyte counts in their blood were reduced at early phases of inflammation. Collectively, monocytes sense the severity of inflammation to decide whether to participate in the inflammatory responses or commit suicide or dysfunction to reduce the tissue damage and ensure the host’s survival.

## 2 Materials and methods

### 2.1 Mice

C57BL/6J (B6) mice were obtained from Japan Slc Inc. (Hamamatsu, Japan). B6.SJL-ptprca (B6.SJL, Strain #: 4007) mice congenic at the CD45 locus (CD45.1^+^CD45.2^−^), CAG-EGFP mice (Strain#: 006567), *Ccr2* ^-/-^ mice (Strain#: 004999) and *Ccr2-creERT2* mice (Strain #: 035229) were obtained from Jackson Laboratories (Bar Harbor, ME, USA). *Casp3^flox/flox^* mice and *Casp7^-/-^* mice were generated as shown in **Supplementary Figures 3A, B**. *Casp3-flox* and *Casp7* Knockout (KO) mice were generated using the CRISPR/Cas system as previously reported (19) with slight modifications. For *Casp3-flox*, two crRNAs were synthesized (Fasmac, Kanagawa, Japan) to target the 5’-CAACAACTCAAGTTAAGTAC-3’ and 5’-GTCTCATCTTGAGGCCAAGG-3’ sequences in introns flanking the *Casp3* exon2 containing the start codon. The plasmid vector used to generate the floxed allele of *Casp3* was constructed with the 5’- and 3’-homology arms of 1500 bases long loxP sequences (5’-ATAACTTCGTATAGCATACATTATACGAAGTTAT-3’) placed at the cleavage sites by Cas9 and restriction sites (SalI and NotI). For *Casp7* KO, two crRNAs targeting the 5’-AAGCAGCCCACAGAACAGCT-3’ sequence in the intron between exons 4 and 5 and the 5’-TGTTTCCTTGCCCCGCCAAG-3’ sequence in the intron between exons 6 and 7 were synthesized (Fasmac) to delete exons 5 and 6. To generate the KO allele with a precisely designed DNA sequence, a 150-mer single-stranded oligodeoxynucleotide (ssODN) was synthesized (5’-CACCTCTTTATAGAGAGAGAAGAAAATGGAGGTTAGCCAACCTCTCTCTGCTGTCACAAAGCAGCCCACAGAACAAAGGGGACCTGGCTCAGTGGGCAAGCTACTTGCAGTACAAGCAATGAGGACCCAAGACCGAATCCCCAGCATCTA-3’ by Hokkaido System Science (Sapporo, Japan). A mixture of Cas9 protein (final concentration of 30 ng/μl, NEB), crRNAs (8.7 ng/μl each), tracrRNA (28.6 ng/μl, Fasmac), the plasmid vector (10 ng/μl) and the ssODN (15 ng/μl) was injected into the pronuclei of C57BL/6J zygotes produced by *in vitro* fertilization. Zygotes surviving after the injection were transferred into the ampullae of the oviducts of pseudopregnant female mice. After natural delivery or caesarean section, newborn pups were genotyped using PCR primer pairs (5’-GTAAGCTAACCGAGCCAATG-3’ and 5’-AGTCAGGTAGATCAGAGGTC-3’ for *Casp3*-flox, 5’-GACGGAGCACACCTTTAATCC-3’ and 5’-CAGTCTGTCGGAATTTGGAGC-3’ for *Casp7* KO), restriction enzymes and Sanger sequencing. Founder mice with the expected genetic modifications were used for further experiments.

### 2.2 Study design for CAR-T cell therapy

We performed an analysis of patients with relapse/refractory diffuse large B cell lymphoma (DLBCL), B-cell lymphoid leukemia (B-ALL), who were treated with CD19-targeted CAR T-cell therapy. We prospectively collected blood samples on CRS patients in Tokyo medical and dental university hospital. Samples were collected from 22 patients between July 2020 and April 2023 and 3 patients were excluded from the analysis of this study according to the reasons shown in **Supplementary Figure 4E**. Nineteen patients were treated with a lymphocyte-depletion regimen (Fludarabine 25 mg/m^2^ + Cyclophosphamide 250 mg/m^2^: Flu+CY) from Day -5 to Day -3 and CD19-specific CAR-T cells (tisagenlecleucel, tisa-cel) (Novartis, Basel, Switzerland) were infused at Day 0. Blood samples were collected before CAR-T cell administration until Day 14. All patients were enrolled in this study with protocols approved by the Institutional Review Board of the Tokyo Medical and Dental University, and written informed consent was obtained from a legally authorized representative as per the Declaration of Helsinki. CRS grading was defined by clinicians blinded to the results of the analysis based on Penn CRS grading (**Supplementary Table 1**) (20). Blood samples were collected in vacutainer tubes containing heparin. Sample tubes were delivered to the laboratory within 4 hours of the sample draw. Peripheral blood mononuclear cells (PBMCs) were purified via density gradient centrifugation and were processed for flow cytometry analysis. Lymphocyte separation solution (d = 1.077, d = 1.119) (Nacalai Tesque Inc., Kyoto, Japan) was used. The blood samples were placed at room temperature for 30–60 min after the blood collection and then kept on ice for all experimental steps until FACS analysis, except for density gradient centrifugation. Plasma samples were stored at -80°C.

Patients’ medical records were reviewed for laboratory data and outcomes. All data were collected from documentation in the medical records. Clinical laboratory studies, including complete blood count (CBC) with differential, C-reactive protein (CRP), lactate dehydrogenase (LDH) and fibrinogen (Fbg) were performed in the Tokyo Medical and Dental University Hospital.

### 2.3 Flow cytometry and cell sorting

After staining with specific antibodies, cells were analyzed using a FACS AriaTM III (BD Bioscience, Franklin Lakes, NJ, USA) and then by FlowJo software (Treestar Inc., Ashland, OR, USA). For sorting of monocytes, BM cells were stained with PE/Cy5-conjugated antibodies against TER119, B220, Ly6G, CD4, CD3e, CD8a (Thermo Fisher Scientific), NK1.1 and CD19 (Biolegend). After washing, cells were incubated with anti-Cy5 microbeads (Miltenyi Biotec, Bergisch Gladbach, Germany) and monocytes were briefly isolated by negative isolation with Auto MACS (Miltenyi Biotec). Monocytes were then stained with a specific antibody and the Ly6C^hi^ monocyte fraction was isolated using a FACS AriaTM III. For counting cell numbers, CountBright™ absolute counting beads (Thermo Fisher Scientific) were used.

### 2.4 Real-time PCR

Total mRNAs were reverse-transcribed to cDNAs and gene expression levels were determined using a Light Cycler 480 and SYBR Green I Master (04707516001, Roche Diagnostics, Basel, Switzerland). The values were normalized according to the expression of β-actin. Specific primers used for real-time PCR are shown in **Supplementary Table 4**.

### 2.5 Quantification of cytokines

To measure cytokine production from monocytes, BM monocytes obtained from wild type (WT) mice before and 12 hours after lipopolysaccharide (LPS, L2880–100mg, Sigma-Aldrich, Saint Louis, MO, USA) treatment (5 mg/kg) were cultured overnight with RPMI-1640 containing 10% FBS in the absence or presence of LPS (100 ng/ml). Supernatants were collected and analyzed using a LEGENDplex™ Mouse Macrophage/Microglia Panel kit (BioLegend, San Diego, CA, USA). *Casp3/7^Δmono^* and their littermate control mice were treated with 20 mg/kg LPS and plasma was collected 2 hours after the LPS injection to evaluate the levels of TNF-α, IL-6, IL-1β and IL-10 using ELISA kits (R&D Systems, Minneapolis, MN, USA and Biolegend). For blood samples of CRS patients, a LEGENDplex™ Human Inflammation Panel 1 kit (Biolegend) was used to measure the levels of cytokines.

### 2.6 Cytological analysis

CD11b^+^Ly6C^hi^Ly6G^-^ monocytes were obtained from the BM 12 hours after treatment (5 mg/kg). Cells were stained with Diff Quik (Sysmex, Kobe, Japan) and the diameters of nuclei in each population were evaluated using Image J.

### 2.7 Evaluation of apoptosis

Cells were obtained from the BM, peripheral blood, and spleen 2, 4, 6 and 12 hours after LPS injection (5 mg/kg). After staining cell surface markers for neutrophils and monocytes, cells were stained with a PE-conjugated anti-Annexin V antibody (Thermo Fisher Scientific, Waltham, MA, USA) and propidium iodide (PI) and were analyzed by flow cytometry. For the detection of cells expressing active caspase-3/7, cells were stained with CellEvent™ Caspase-3/7 Detection Reagents (Thermo Fisher Scientific) after the cell surface marker staining. For detection of cells expressing active caspase-1, cells were stained with a FAM-FLICA^(R)^ Caspase 1 Assay Kit (Immunochemistry Technologies, Davis, CA, USA) after the cell surface marker staining.

### 2.8 *In vitro* stimulation of BM cells

Monocytes, neutrophils, and B cells isolated from naïve WT mice were cultured with LPS (10–1000 ng/ml) and/or cytokines (50 ng/ml, Biolegend) at 37°C for 3 hours. For caspase-blockade, cells were pre-cultured with inhibitors for Caspase-1 (Z-YVAD-FMK), Caspase-3 (Z-DEVD-FMK), Caspase-8 (Z-IETD-FMK) or Caspase-9 (Z-LEHD-FMK) (1–100 μM) (R&D Systems) before the stimulation. After stimulation, live cell numbers were counted by flow cytometry using CountBright™ absolute counting beads (Thermo Fisher Scientific).

### 2.9 Cecal ligation and puncture model

Mice were anesthetized by intraperitoneal injection of an anesthetic mixture of medetomidine, midazolam and butorphanol. The abdomen of each mouse was then shaved and a laparotomy was performed. The cecum was exposed and tightly ligated 1.0 cm from the distal end. The ligated cecum was then perforated twice with a 26G or 19G needle. The cecum was returned to the peritoneal cavity after gentle squeezing to extrude a small amount of feces from the perforated sites. The peritoneum was sutured, and the skin was closed using a clip. Twelve hours after CLP induction, the mice were sacrificed and the frequencies and absolute numbers of neutrophils and monocytes in the BM and the peritoneal cavity were determined using flow cytometry.

### 2.10 Cytokine administration

Carrier-free recombinant mouse TNF-α and IFN-γ (10 μg/mouse each) (Biolegend, endotoxin level: <0.1 EU/µg protein) were mixed and administered intraperitoneally to WT C57BL/6J mice after which the numbers, PI^+^ dead cell ratio, and active caspase3/7-expressing apoptotic cell ratio in monocytes and neutrophils obtained from the BM and peripheral blood were evaluated 6 hours after the cytokine treatment.

### 2.11 Intra-BM transplantation

Mice were anesthetized by an intraperitoneal injection of an anesthetic mixture of medetomidine, midazolam and butorphanol, after which monocytes obtained from the BM of CAG-EGFP mice were transplanted into the tibias of recipient B6.SJL mice. After the intra-BM transplantation, mice were quickly recovered by an injection of atipamezole. The mice were then treated with LPS (5 mg/kg) 1 hour after the transplantation. Eighteen hours after treatment, the distribution, expression of monocyte markers and cell death of donor cells were analyzed using FACS. To examine the apoptosis in monocytes, monocytes sorted from the BM of naïve TLR4-deficient or sufficient mice were transplanted into the BM of B6.SJL mice. Then, LPS (5 mg/kg) was intraperitoneally injected into the recipient mice and the apoptotic cell ratio was determined in the BM donor monocytes 6 hours after the LPS-treatment.

### 2.12 Transwell migration assay

To evaluate the migration capacity of monocytes toward CCL2, Transwell migration assays (3-μm pores, Corning, Pittsburgh, PA, USA, #3402) were performed. Monocytes (3×10^5^ cells) obtained from the BM before and after LPS treatment (5 mg/kg) were placed in the upper chamber and different doses of recombinant CCL2 (0, 2.5, 10, 50 ng/ml) (Biolegend) were added in the lower chamber. The cells were then cultured 12 hours at 37˚C with 5% CO_2_. Cells that had migrated from the lower chamber were counted by FACS with cell counting beads (Thermo Fisher Scientific).

### 2.13 Western blot analysis

Western blotting was performed as previously described (21). *Casp3/7^Δmono^* mice were treated with 100 mg/kg tamoxifen intraperitoneally for 5 days. Two days after the last injection of tamoxifen, monocytes (2–3×10^5^ cells) were sorted from the BM of WT mice and of *Casp3/7^Δmono^* mice and were lysed in lysis buffer containing 1% Triton X-100, 20 mM Tris–HCl (pH 7.5), 150 mM NaCl, 1 mM EDTA, 1 mM sodium orthovanadate, 1 mM phenylmethylsulfonyl fluoride, 10 μg/ml aprotinin and 10 μg/ml leupeptin. The lysates were incubated on ice for 15 minutes and the supernatants were collected after centrifugation at 15,000 rpm, 4 ˚C for 20 min. The supernatants were mixed with 1 volume of 2x Laemmli sample buffer and were separated by electrophoresis on SDS-PAGE gels (Biorad, Hercules, CA, USA). The proteins were transferred to Immobilon P membranes (Millipore, Darmstadt, Germany) using a wet transfer method. Western blotting was performed using specific antibodies against β-actin (Sigma-Aldrich), Caspase3 (Cell Signaling Technology, Danvers, MA, USA, #14220T) and Caspase7 (Cell Signaling #12827T) and the proteins detected by those antibodies were visualized using a chemiluminescence kit (Western Lightning Plus-ECL, PerkinElmer, Waltham, MA, USA).

### 2.14 Statistical analysis

Statistical analyses were performed using Microsoft Excel or Prism software version 3 (GraphPad, San Diego, CA, USA). A two-tailed Student’s t-test was used for statistical analyses of two-group comparisons. Multigroup comparisons were performed using one-way analysis of variance (ANOVA) followed by the Tukey–Kramer multiple comparisons test. The criterion of significance was set at p < 0.05. Results are expressed as means ± standard error of the mean (SEM). For analysis of survival of the high dose LPS challenge, each group was subjected to Kaplan-Meier analysis and comparisons were performed using the log-rank test. CRS grading was defined by clinicians blinded to the results of analysis based on Penn CRS grading (**Supplementary Table 1**). Samples from 3 patients were excluded from the analysis as shown in **Supplementary Figure 4E**. Randomization of the groups was not performed. No statistical methods were used to estimate sample size.

## 3 Results

### 3.1 Disappearance of monocytes at early phase of systemic inflammation

**Figure 1:**
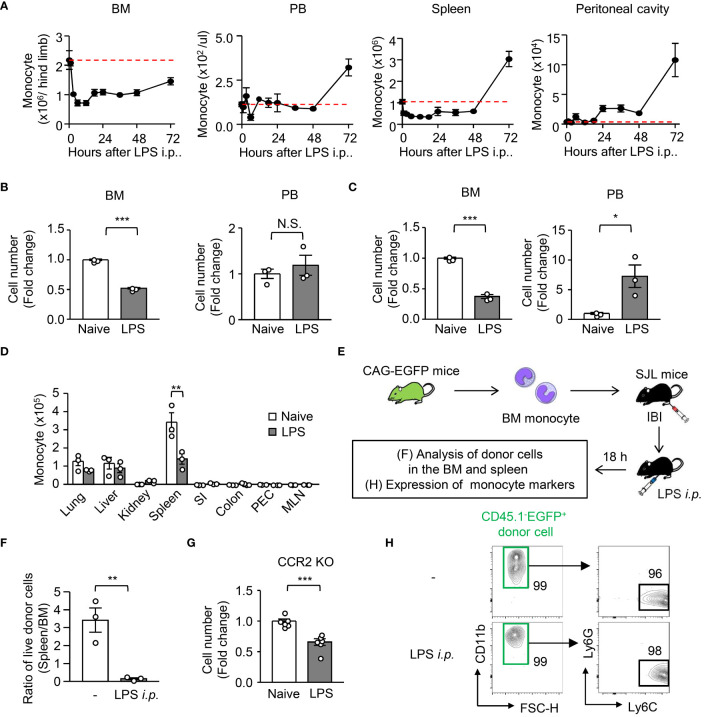
Kinetic analysis of monocytes and neutrophils during LPS-induced inflammation. LPS (5 mg/kg) was intraperitoneally injected into WT C57BL/6J mice to mimic systemic inflammation caused by a systemic bacterial infection. **(A)** Kinetics of monocytes (Mo) in the BM, peripheral blood (PB), spleen and peritoneal cavity from 0 to 72 hours after treatment with LPS; red dotted lines indicate monocyte numbers at naïve conditions. **(B–D)** Numbers of monocytes **(B, D)** and neutrophils **(C)** in the BM and PB **(B, C)** and/or lung, liver, kidney, spleen, small intestine (SI), colon, peritoneal cavity (PEC) and mesenteric lymph node (MLN) **(D)** 12 hours after LPS treatment; n=3 each group. **(E, F, H)** Experimental strategy for intra-BM injection of monocytes obtained from CAG-EGFP mice **(E)**. Monocytes obtained from the BM of naïve CAG-EGFP mice were transferred into the BM of B6.SJL mice after which the mice received LPS treatment. The ratio of monocyte number between spleen and BM **(F)** and the expression of monocyte markers **(H)** were examined 18 hours after LPS treatment; n=3 each group. The numbers on the FCM plots indicate the frequencies of the gated populations. **(G)** Monocyte number in the BM of CCR2-deficient mice before and 12 hours after LPS treatment; n=3 each group. *p<0.05, **p<0.01, ***p<0.001, N.S., not significantly different (Student’s t-test). Data are representative of two independent experiments (error bars, SEM).

To examine the kinetics of monocytes in acute systemic inflammation, we intraperitoneally administered a sublethal dosage of LPS (5 mg/kg) to WT C57BL/6 mice to induce sepsis. After LPS treatment, the upregulation of chemoattractants for monocytes (*Ccl2, Ccl3, Ccl4, Ccl6, Ccl7* and *Ccl8*) and neutrophils (*Cxcl1* and *Cxcl2*) was examined in various peripheral organs (**Supplementary Figure 1A**). Consistent with previous reports, major monocyte and neutrophil chemoattractants such as CCL2, CCL7, and CXCL1/2 were substantially elevated (**Supplementary Figure 1A**), which are driven by common transcription factors NF-κB and/or AP-1 both in mice and in humans (22–24). We then examined the kinetics of monocytes in the BM, peripheral blood, spleen, and peritoneal cavity of mice after LPS treatment and found that about half of classical monocytes (hereafter referred to as “monocyte”) and neutrophils were lost in the BM within 12 hours after sepsis induction (**Figures 1A–C**; gating strategy is shown in **Supplementary Figure 1B**). Interestingly, the increase of monocytes was not observed in the blood and spleen until 72 hours after LPS treatment (**Figures 1A, B**), although neutrophils were greatly increased in the peripheral blood 12 hours after sepsis induction (**Figure 1C**). Thus, we investigated where the monocytes that were lost in the BM went. However, LPS injection did not increase the number of monocytes in most peripheral organs at 12 hours after treatment (**Figure 1D**). In some organs, such as the peritoneal cavity and kidney, an increase of monocytes was observed within 12 or 24 hours after LPS treatment (**Figures 1A, D**). Consistent with previous reports (25, 26), monocytes accumulated transiently in the lungs 2 hours after the injection of LPS. However, those effects were too small to explain the decrease of monocytes in the BM (**Figures 1A, D**, **Supplementary Figure 1C**). We transferred Ly6C^hi^ monocytes from CAG-EGFP mice (CD45.2) into the BM of B6.SJL mice (CD45.1) and examined the donor-derived monocytes in the BM and spleen 12 hours after LPS treatment (**Figure 1E**). Compared to naïve conditions, the LPS treatment significantly decreased the ratio of splenic monocytes/BM monocytes (**Figure 1F**), suggesting that BM monocytes do not distribute to the periphery at the early stage of LPS-induced sepsis. To further demonstrate that the LPS-induced monocyte reduction in the BM was unlikely due to the egress of monocytes from the BM, we induced LPS-induced sepsis in CCR2-deficient mice (**Figure 1G**). As the decrease of monocytes in the BM was observed even in CCR2-deficient mice 12 hours after LPS treatment (**Figure 1G**), the egress of monocytes from the BM seems not to be a cause of the monocyte reduction in the BM at the early stage of sepsis. To further examine whether LPS treatment altered the expression of monocyte markers, which would make monocyte identification difficult, we transferred BM-derived monocytes from CAG-EGFP mice into the BM of recipient mice and examined the expression of monocyte markers 12 hours after LPS treatment (**Figures 1E, H**). The expression of CD11b and Ly6C was maintained and Ly6G was not expressed in the donor-derived monocytes regardless of LPS-injection (**Figure 1H**), ruling out the possible alteration of monocyte markers.

### 3.2 Monocytes are eliminated by apoptosis early after systemic inflammation

**Figure 2:**
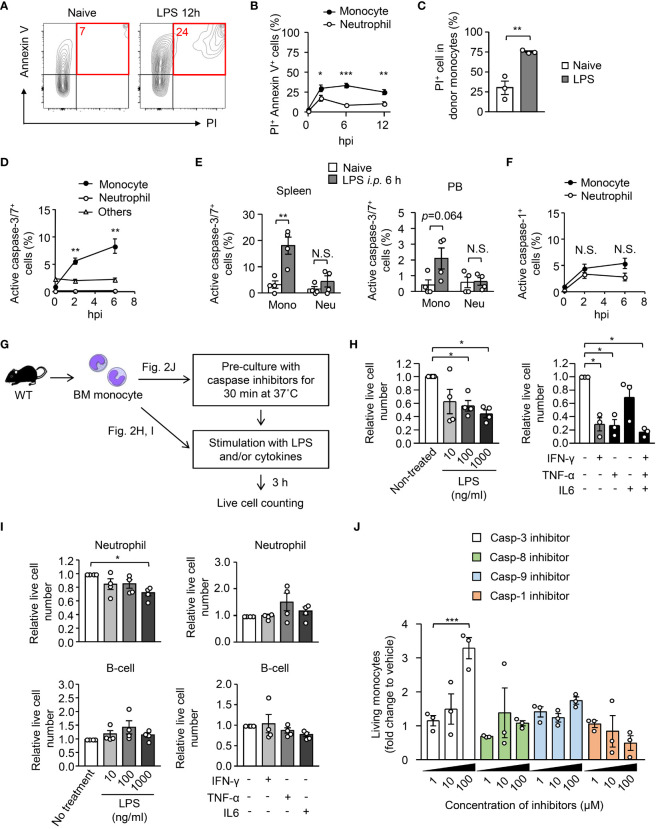
Exclusion of monocytes by apoptosis early after systemic inflammation. **(A, B)** Evaluation of cell death of BM monocytes before and after intraperitoneal injection of LPS (5 mg/kg). BM cells were obtained before and 6 or 12 hours after LPS treatment and the frequencies of the PI^+^ AnnexinV^+^ fraction were measured using FACS; representative FACS plots are shown in **(A)**. **(C)** EGFP^+^ monocytes were transferred to B6.SJL mice as shown in **Figure 1E** and the frequency of PI^+^ dead cells was examined in donor monocytes 18 hours after LPS treatment; n=4 per group. **(D, E)** Frequencies of apoptotic cells in monocytes obtained from the BM **(D)**, spleen and PB **(E)** were evaluated by counting active caspase-3/7-expressing cells; n=4 per group for **(D, E)**. **(F)** Frequencies of active caspase-1-expressing cells in BM monocytes before and after LPS administration; n=4 per group. **(G–I)** Induction of apoptosis in monocytes by *ex vivo* stimulation with LPS or cytokines. Monocytes **(H)**, neutrophils and B cells **(I)** obtained from the BM of naïve WT mice were stimulated with LPS or indicated cytokines for 3 hours, and surviving cell numbers were counted by FACS with cell counting beads; n=4 per group. The experimental strategy is shown in **(G)**. **(J)** BM monocytes obtained from naïve WT mice were pre-cultured in the presence of caspase inhibitors at various concentrations as indicated for 30 minutes and then were stimulated with LPS (10 ng/ml) for 3 hours. Surviving cell numbers were examined by FACS with cell counting beads. *p<0.05, **p<0.01, ***p<0.001, N.S., not significantly different (one-way ANOVA). Data are representative of two independent experiments (error bars, SEM).

To examine the reason for the disappearance of monocytes in peripheral organs after LPS treatment, we evaluated cell death in monocytes and neutrophils 12 hours after LPS treatment. Staining with propidium iodide (PI) and Annexin V revealed that the frequency of dead cells (PI^+^ Annexin V^+^) in monocytes was increased immediately after LPS treatment (**Figure 2A**), and the increase of cell death in monocytes was significantly higher than that in neutrophils (**Figure 2B**). When monocytes isolated from the BM of CAG-EGFP mice were transferred into the BM of recipient mice (as shown in **Figure 1E**), the frequency of PI^+^ dead cells was increased in donor monocytes after LPS treatment (**Figure 2C**). Staining for active caspase-3/7 demonstrated that apoptosis is selectively induced in monocytes, but not in neutrophils or other leukocytes in the BM, spleen and peripheral blood after LPS treatment (**Figures 2D, E**), which suggests that monocyte apoptosis was induced in both the BM and the periphery. In contrast, the frequencies of active caspase-1-expressing cells were comparable between monocytes and neutrophils after LPS treatment (**Figure 2F**), suggesting that pyroptosis could not explain the selective disappearance of monocytes in peripheral organs after sepsis induction.

We next examined whether inflammatory stimuli cause the cell death of monocytes *in vitro*. We isolated Ly6C^hi^ monocytes from the BM of naïve WT mice and cultured them with LPS or with inflammatory cytokines for 3 hours (**Figure 2G**) and found that LPS and inflammatory cytokines such as IFN-γ and TNF-α but not IL-6, quickly decreased the number of live monocytes (**Figure 2H**). In contrast, the impact of the stimulation with LPS or with cytokines in the cell death of neutrophils and B cells was much lower than that of monocytes (**Figure 2I**), which suggests that the monocyte-specific cell death program is driven by infection/inflammation. Consistent with the low induction of apoptosis in B cells, we recently showed that the number of B cells was not decreased in the BM within 12 hours after a high dose LPS injection (27). To demonstrate how monocytes were killed by inflammatory stimuli, we pre-cultured monocytes in the presence of inhibitors for caspases-3, 8, 9 or 1 for 30 minutes and then stimulated them with LPS. Live cell counts revealed that only the caspase-3 inhibitor suppressed the cell death (**Figure 2J**), which indicates that monocytes are eliminated by apoptosis. Taken together, microbial and/or inflammatory signals eliminate monocytes by inducing apoptosis early after the onset of sepsis.

### 3.3 Functional alteration of monocytes in the BM during systemic inflammation

**Figure 3:**
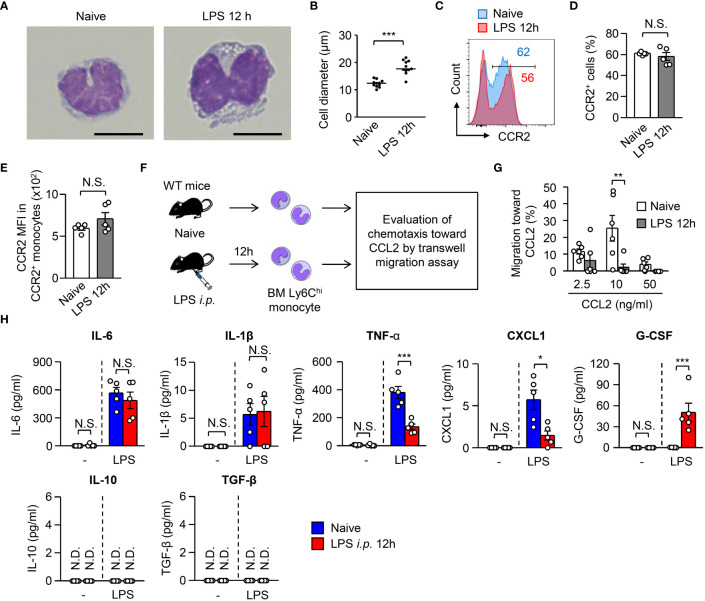
Monocytes surviving in the BM after LPS treatment lose their functions. **(A, B)** Morphology of monocytes obtained from the BM of WT C57BL/6 mice before and 12 hours after LPS treatment (5 mg/kg). Representative images and the maximum diameter of BM monocytes are shown in **(A, B)**, respectively; scale bars indicate 5 μm. **(C–E)** Expression of CCR2 on monocytes obtained from the BM of WT C57BL/6 mice before and 12 hours after LPS treatment (5 mg/kg). Representative FACS plots are shown in **(C)** and statistical analysis of the frequencies of CCR2^+^ cells **(D)** and the CCR2 mean fluorescence intensity (MFI) **(E)** of BM monocytes is shown in **(D)**; n=5 per group. **(F, G)** Transwell migration assay for BM monocytes. Monocytes were obtained from the BM of naïve or LPS-treated WT C57BL/6 mice (3×10^5^ cells/well) and were added to the upper chamber with the indicated concentration of recombinant mouse CCL2 added in the lower chamber. The cells were then incubated at 37˚C overnight, after which the number of migrated monocytes was counted by FACS with cell-counting beads. The experimental strategy is shown in **(E)** and statistical analysis of specific migration toward CCL2 is shown in **(F)**; n=6 per group. **(H)** Cytokine production by monocytes obtained from the BM of naïve or LPS-treated mice. BM monocytes were obtained from WT mice before and 12 hours after LPS treatment (5 mg/kg) and were cultured (5×10^5^ cells/ml) in the presence of LPS (100 ng/ml) for 24 hours. N.S., not significantly different. Supernatants were collected and the production of cytokines was evaluated using Legendplex. *p<0.05, **p<0.01, ***p<0.001, N.S., not significantly different; N.D., not detectable [Student t-test **(D, E)** and one-way ANOVA **(G)**]. Data are representative of two independent experiments **(A–E)** or pooled from two independent experiments **(G)** (error bars, SEM).

Half of the monocytes were eliminated by apoptosis early after sepsis, meaning that the other half of the monocytes survived (**Figures 1A, B**). We characterized the surviving monocytes in the BM. The monocytes from LPS-treated mice and from naïve monocytes showed kidney-shaped nuclei, a representative monocyte morphology (**Figure 3A**). However, the LPS-treated monocytes were significantly larger in size than the naïve monocytes (**Figure 3B**). Monocyte egress from the BM is dependent on CCR2, a receptor for CCL2, during infection (28). The BM monocytes from LPS-treated mice maintained their CCR2 expression even after LPS treatment (**Figures 3C–E**). However, they showed an impaired migration ability toward CCL2 compared to BM monocytes from naïve mice (**Figures 3F, G**), which suggested that surviving monocytes in the BM after sepsis had lost their capacity to migrate out of the BM.

M-CSF-mediated signaling promotes monocyte differentiation into macrophages, and LPS treatment induces ADAM17-dependent shedding of CD115, a receptor for M-CSF (29). In this context, CD115 expression on the surface of BM monocytes was largely abolished within 3 hours after LPS treatment (**Supplementary Figure 2A**). To examine the differentiation capacity of monocytes *in vivo*, we obtained BM monocytes before and after LPS treatment and transferred them into the peritoneal cavity of naïve B6.SJL mice, after which flow cytometry was used to examine the generation of F4/80^+^Ly6C^-^ macrophages from F4/80^-^Ly6C^+^ monocytes (**Supplementary Figure 2B**). As expected, the monocytes obtained from LPS-treated mice showed impaired differentiation into macrophages (**Supplementary Figures 2C, D**).

Monocytes act as a source of cytokines during infections (17, 28). Thus, we evaluated the cytokine production ability of BM monocytes from naïve and from LPS-treated mice (**Figure 3H**, **Supplementary Figure 2E**) and found that BM monocytes from LPS-treated mice showed significantly lower production of TNF-α, a representative cytokine involved in sepsis induction, and CXCL1 (**Figure 3H**). In contrast, monocytes from LPS-treated mice, but not from naïve mice, were able to produce G-CSF (**Figure 3H**).

Together with the defects in migration, differentiation, and cytokine production, the surviving monocytes in the BM after LPS treatment are dysfunctional in terms of sepsis induction.

### 3.4 Severe inflammation prevents an increase of monocytes in the periphery

**Figure 4:**
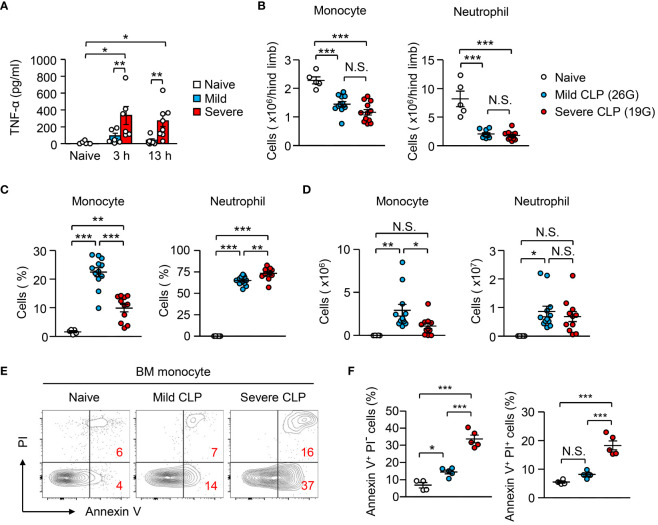
Peripheral monocyte supply is dependent on the magnitude of inflammation. **(A–F)** Mild and severe CLP was performed in WT mice using 26G and 19G needles, respectively; n=5 for naïve, n=12 for mild CLP and n=11 for severe CLP. **(A)** Levels of plasma TNF-α were evaluated 3 or 13 hours after the induction of mild or severe CLP. **(B–D)** Number **(B, D)** and frequency **(C)** of monocytes and neutrophils in the BM **(B)** and peritoneal cavity **(C, D)** 13 hours after CLP-induction. **(E, F)** Frequencies of early apoptotic cells (Annexin V^+^ PI^-^) and dead cells (Annexin V^+^ PI^+^) in BM monocytes before and 13 hours after CLP induction; representative FACS plots are shown in **(E)**. *p<0.05, **p<0.01, ***p<0.001, N.S., not significantly different [one-way ANOVA **(A–D, F)**]. Data are pooled from two **(A)** or five **(B–F)** independent experiments (error bars, SEM).

A previous report suggested that administration of high-dose LPS does not increase circulating monocytes, although low-dose LPS does that (26). However, the mechanism and physiological meaning of that are unknown. In this context, our results from monocyte cultures revealed that the stronger the inflammatory stimulus, the greater the apoptosis of monocytes (**Figure 2H**), which implies that severe inflammation more efficiently suppresses monocyte appearance *in vivo*. Using a cecal ligation and puncture (CLP) infection model, we induced mild and severe sepsis with 26G and 19G needles, respectively. Severe sepsis-induced mice showed significantly higher plasma levels of TNF-α compared to mild sepsis-induced mice (**Figure 4A**). In both settings, the numbers of monocytes and neutrophils were similarly decreased in the BM within 12 hours after CLP-induction (**Figure 4B**). However, the frequency and number of monocytes that migrated into the peritoneal cavity were significantly higher in mice with mild sepsis compared to mice with severe sepsis (**Figures 4C, D**). In contrast, the number of neutrophils was comparable between the severe and mild CLP settings (**Figures 4C, D**). As we observed in the LPS injection model (**Figures 2D, E**), the apoptotic cell ratio in BM monocytes of severe sepsis-induced mice was significantly higher than that of mild sepsis-induced mice (**Figures 4E, F**). Since it has been reported that the injection of low-dose LPS (20 ng/body) increases monocytes in the periphery (26), we also tested the impact of treatment with low-dose (20 ng/body) and high-dose (100 μg/body) LPS in the apoptosis of monocytes (**Supplementary Figure 2F**). Consistent with our results with the CLP model, low-dose LPS injection did not induce apoptosis, although high-dose LPS injection did induce apoptosis. Thus, monocytes are eliminated by apoptosis only when the inflammation is excessive.

### 3.5 Inhibition of apoptosis in monocytes exacerbates systemic inflammation during sepsis

**Figure 5:**
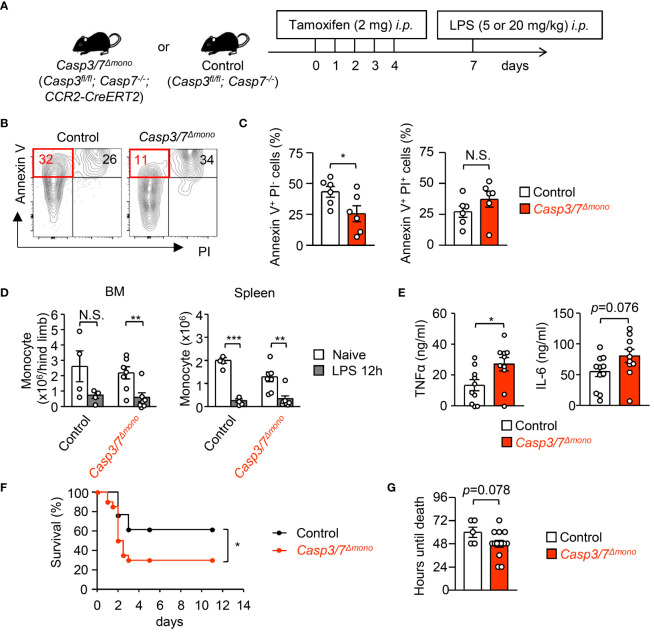
Casp3/7-deficiency in monocytes exacerbates systemic inflammation during sepsis. **(A)** Experimental strategy with *Casp3/7^Δmono^* mice (*Casp3^fl/fl^;Casp7^-/-^;CCR2-CreERT2*) and their littermate control (*Casp3^fl/fl^;Casp7^-/-^*) mice. **(B, C)** Frequencies of early apoptotic cells (Annexin V^+^ PI^-^) and dead cells (Annexin V^+^ PI^+^) in monocytes obtained from the BM of *Casp3/7^Δmono^* and control mice 12 hours after LPS treatment (5 mg/kg); n=6 per group. **(D)** Numbers of monocytes in the BM and spleens of *Casp3/7^Δmono^* and control mice before and 12 hours after LPS treatment (5 mg/kg); n=4 for control, n=7 for *Casp3/7^Δmono^*. **(E)** Concentration of TNF-α and IL-6 in the plasma of *Casp3/7^Δmono^* mice and control mice 2 hours after LPS treatment (5 mg/kg); n=10 per group. **(F, G)** LPS (20 mg/kg) was administered to *Casp3/7^Δmono^* mice and control mice and their mortality was monitored for 11 days. Kaplan-Meier survival curve and the life times of mice are shown in **(F, G)**, respectively; n=20 for *Casp3/7^Δmono^* mice, n=13 for control mice. *p<0.05, **p<0.01, ***p<0.001, N.S., not significantly different [Student’s t-test **(C, E, G)**, one-way ANOVA **(D)** or log-rank test **(F)**]; data are pooled from two **(B–E)** or three **(F, G)** independent experiments (error bars, SEM).

A CCR2-deficiency improved the severity of sepsis and a supply of peripheral monocytes restores sepsis in CCR2-deficient mice (17), suggesting that the existence of monocytes in the periphery enhances systemic inflammation. To further examine if the apoptosis-mediated reduction of monocytes alters the magnitude of inflammation, we generated *Casp7^-/-^;Casp3^flox/flox^;CCR2-CreERT2* (*Casp3/7^Δmono^*) mice (**Supplementary Figures 3A–C**). We injected tamoxifen (2 mg/mouse/day) into *Casp3/7* ^Δmono^ mice and their littermate control mice (*Casp3^fl/fl^;Casp7^-/-^* mice) for 5 consecutive days (**Figure 5A**) and confirmed that monocytes from *Casp3/7* ^Δmono^ mice after that treatment lacked Caspase-3 and 7 by western blotting (**Supplementary Figure 3D**). The frequency of Annexin^+^ PI^-^ early apoptotic cells was also successfully decreased in monocytes by the genetic ablation of *Casp3/7* after LPS treatment (**Figures 5B, C**). On the other hand, the frequency of the Annexin-V^+^ PI^+^ fraction, which is a mixture of necrotic cells and late apoptotic cells, was not significantly changed (**Figures 5B, C**), which suggests that the monocyte death was induced by non-apoptotic cell death such as necrosis in *Casp3/7* ^Δmono^ mice. Consistently, the blockade of apoptosis results in the induction of necroptosis (30–32). Indeed, the number of monocytes was equally reduced in control and in *Casp3/7* ^Δmono^ mice (**Figure 5D**). In this context, there was no significant difference in the frequency of monocytes expressing active caspase-1, an indicator of pyroptosis, between control and *Casp3/7* ^Δmono^ mice (**Supplementary Figure 3E**). Collectively, these results imply the necrosis-mediated clearance of monocytes in *Casp3/7* ^Δmono^ mice. To evaluate the importance of monocyte death by apoptosis in fine-tuning the magnitude of inflammation, we examined blood cytokine levels 2 hours after LPS treatment and found that the levels of TNF-α and IL-6 were increased in *Casp3/7* ^Δmono^ mice compared to control mice (**Figure 5E**). In contrast, the levels of IL-1β and IL-10 were comparable between the two groups (**Supplementary Figure 3F**). Reflecting the severe inflammation, the weight and size of the spleens in *Casp3/7* ^Δmono^ mice were slightly increased (**Supplementary Figure 3G**). In addition, the mortality of *Casp3/7* ^Δmono^ mice was significantly increased compared to control mice during sepsis induced by treatment with a lethal dosage of LPS (20 mg/kg) (**Figures 5F, G**). These results suggested that the apoptosis-dependent reduction of monocytes is crucial for fine-tuning excessive inflammation during sepsis.

### 3.6 Cytokine-mediated stimulation is sufficient to induce monocyte disappearance in periphery

**Figure 6:**
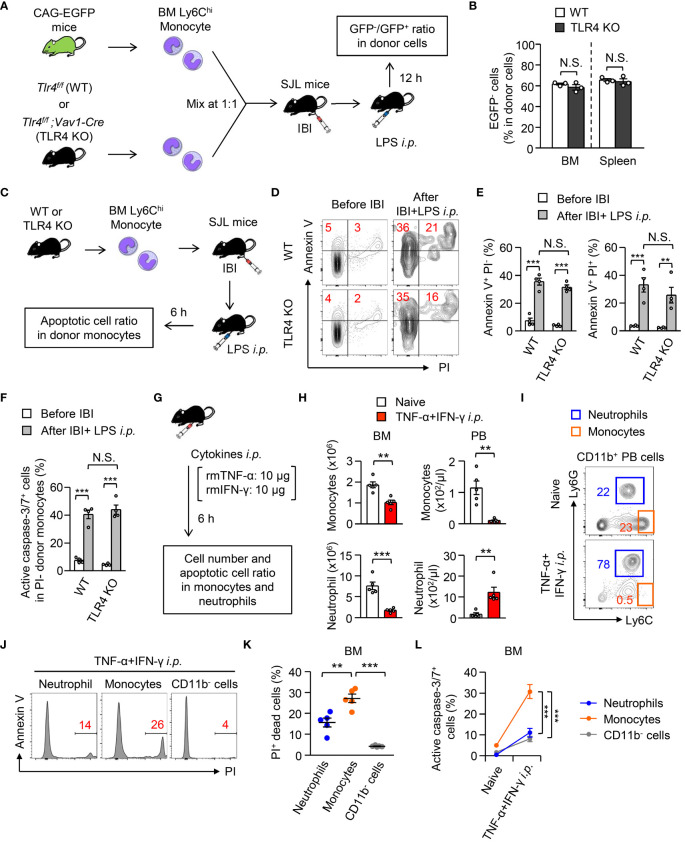
Importance of TLR4-mediated signaling to trigger monocyte cell death during systemic inflammation. **(A, B)** Experimental strategy for co-IBI of TLR4-sufficient or TLR4-deficient monocytes and EGFP-expressing monocytes **(A)**. Monocytes were obtained from the BM of naïve *Tlr4^flox/flox^;Vav1-Cre* mice (TLR4 KO) or their littermate control (*Tlr4^flox/flox^*; WT) mice and were mixed with monocytes obtained from naïve CAG-EGFP mice at a 1:1 ratio. The mixed monocytes were transferred into the BM of recipient SJL mice and the recipient mice received an intraperitoneal injection of LPS (5 mg/kg). Twelve hours after treatment, the ratio of GFP^-^ and GFP^+^ cells in CD45.2^+^CD45.1^-^ donor cells was examined **(B)**. **(C–F)** BM monocytes obtained from TLR4 KO or control mice were injected into the BM of B6.SJL mice after which the mice were treated with LPS (5 mg/kg) as shown in **(C)**. Six hours after the treatment, the frequencies of Annexin V^+^PI^+^ dead cells, Annexin V^+^PI^-^ early apoptotic cells **(D, E)**, and active caspase3/7-expressing cells **(F)** in donor monocytes were examined. Representative FACS plots are shown in **(D)**. **(G–L)** A mixture of recombinant mouse TNF-α and IFN-γ (10 μg/mouse each) was injected intraperitoneally to WT B6 mice and the numbers **(H)**, dead cell ratio **(J, K)**, and active caspase3/7-expressing cell ratio **(L)** of monocytes, neutrophils and/or CD11b^-^ cells in the BM and PB were examined 6 hours after the cytokine administration. The experimental strategy is shown in **(G)**. Representative FACS plots for CD11b^+^ cells in the PB and monocytes and neutrophils in the BM are shown in **(I, J)**, respectively. **p<0.01, ***p<0.001, N.S., not significantly different [Student *t*-test **(B, H)**, one-way ANOVA **(D, E, K, L)**]. Data are pooled from two independent experiments (Error bars, SEM).

In contrast to immune paralysis, which is induced at a later phase of sepsis (33), we showed that monocyte apoptosis at an early stage of sepsis protects the host from excessive inflammation, implying that this mechanism might work independently of microbial stimuli. To examine that hypothesis, TLR4-deficient or TLR4-sufficient monocytes were mixed with monocytes from TLR4-sufficient CAG-EGFP mice at a 1:1 ratio and were directly transplanted into the BM of B6.SJL mice. When we examined the ratio between EGFP^+^ and EGFP^-^ donor (TLR4-deficient or TLR4-sufficient) monocytes 12 hours after LPS treatment (**Figure 6A**), the TLR4-deficiency did not alter that ratio (**Figure 6B**). Next, we administrated LPS (5 mg/kg) into SJL mice that received an intra-BM transplantation of WT or TLR4-deficient monocytes (**Figure 6C**). Importantly, the TLR4-deficiency did not alter the ratio of Annexin V^+^PI^+^ dead cells, Annexin V^+^ PI^-^ early apoptotic cells (**Figures 6D, E**) or active caspase3/7-expressing cells in donor monocytes (**Figure 6F**). These results suggested that TLR4-mediated signaling is not required to trigger monocyte apoptosis *in vivo* and implied the importance of pro-inflammatory cytokine-mediated signaling. To directly test this *in vivo*, we treated WT mice with recombinant TNF-α and IFN-γ as reported previously (34) (**Figure 6G**), which caused monocyte cell death *in vitro* (**Figure 2H**), and found that the numbers of monocytes and neutrophils were significantly reduced in the BM 6 hours after the treatment (**Figure 6H**). Importantly, monocytes simultaneously disappeared from the peripheral blood, while the number of neutrophils was greatly increased (**Figures 6H, I**). At this time, the increases in dead cells, which were evaluated by PI staining, and caspase3/7-expressing cells were pronounced in monocytes compared with neutrophils and CD11b^-^ cells in the BM (**Figures 6J–L**). Collectively, these results suggested that a microbial stimulus is not required while a pro-inflammatory cytokine stimulation is sufficient to induce monocyte apoptosis, which resulted in the selective disappearance of monocytes in the periphery.

### 3.7 The monocyte disappearance during human cytokine release syndrome

**Figure 7:**
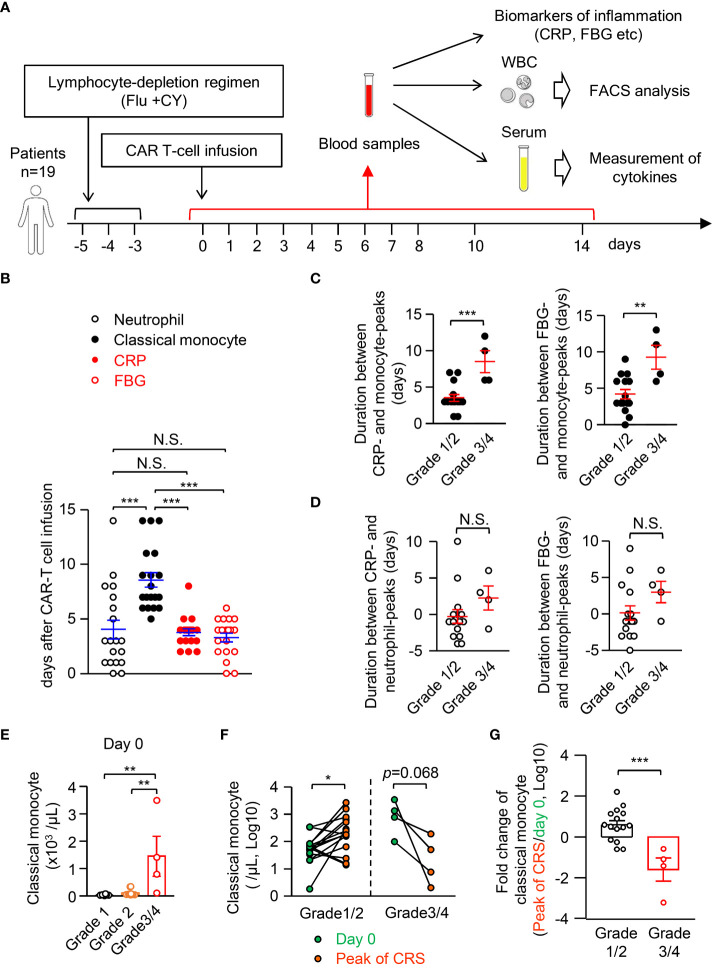
Classical monocytes disappear in the peripheral blood during human CRS. **(A)** Experimental strategy for monitoring inflammation biomarkers, numbers of leukocytes and levels of cytokines in blood samples of CRS patients who received CAR-T cell therapy; n=19. **(B)** The peak day for cell counts of neutrophils and classical monocytes, CRP and FBG in the PB of CRS patients. **(C, D)** Duration between CRP or FBG and classical monocytes **(C)** or neutrophils **(D)**. **(E)** Cell count of classical monocytes in the blood of patients of each grade of CRS at day 0. **(F)** Number of classical monocytes in the blood at day 0 and at the peak of CRS. **(G)** Fold changes of classical monocytes in the blood from day 0 to the peak of CRP are shown. *p<0.05, **p<0.01, ***p<0.001, N.S., not significantly different [Student’s t-test **(C, D, F, G)** or one-way ANOVA **(B, E)**]. Error bars, SEM.

As pro-inflammatory cytokines are sufficient to induce apoptosis-mediated monocyte disappearance in mice (**Figure 6**), we aimed to determine whether it is also observed in human patients during pathogen-unrelated systemic inflammation. To this end, we focused on cytokine-release syndrome (CRS), which is caused by the release of large amounts of cytokines accompanying an excessive immune response not only during systemic infection but also during CAR-T cell immunotherapy. It should be emphasized that CAR-T cell administration has the advantage of monitoring CRS from the starting point of inflammation, which is not feasible in patients with infectious diseases or other inflammatory diseases because the symptoms are already present at the time the patient is seen in the hospital. In the CAR-T cell-induced CRS model with humanized mice and human CAR-T cells, human monocytes act as a major source of cytokines (35). We monitored the kinetics of CD14^+^CD16^low^ classical monocytes, neutrophils, c-reactive protein (CRP), and fibrinogen (FBG) in the blood of 19 patients until day 14 after CAR-T cell administration (**Figure 7A**, the gating strategy of classical monocytes and neutrophils is shown in **Supplementary Figure 4A**). The severity of CRS was graded based on the Penn grading scale (**Supplementary Table 1**). All patients who received CAR-T cell therapy developed CRS (n=19), but only four patients developed severe CRS (grade 3 and 4, 15.8%). Patient characteristics with each CRS grade were shown in **Supplementary Table 2**. The peak of blood neutrophil count almost coincided with the peaks of the inflammation markers CRP and FBG (**Figure 7B**). In contrast, the peak of classical monocyte count was later than the peaks of CRP and FBG, implying the suppression of monocyte appearance in the periphery during systemic inflammation in humans. In this context, the duration between the peak of monocytes, but not neutrophils, and the peak of CRP or FBG became larger in patients with severe CRS (grades 3 and 4) compared to those with relatively mild CRS (grades 1 and 2) (**Figures 7C, D**). Thus, the more severe the inflammation, the more delayed the appearance of classical monocytes in the blood during CRS. Related to this, patients with higher counts of classical monocytes before CAR-T therapy had a more severe CRS after receiving CAR-T therapy (day 0) (**Figure 7E**). On the other hand, the blood levels of cytokines, neutrophils and clinical indicators of inflammation at day 0 did not affect the severity of CRS (**Supplementary Figures 4B–D**; **Supplementary Table 3**), implying that the counts of classical monocytes in the blood before starting CAR-T therapy may serve as a biomarker to predict the severity of subsequent CRS after the treatment. Importantly, compared to day 0, the counts of monocytes in the blood were significantly increased at the peak of CRS in grade 1/2 patients, whereas grade 3/4 patients showed a decrease of monocytes in their blood at that time (**Figures 7F, G**). Those results obtained from patients are consistent with results obtained from mice (**Figure 4**), and the magnitude of inflammation is critical for changing monocyte behavior both in mice and in humans.

## 4 Discussion

Monocytes are a unique population of cells that have the potential to differentiate into multiple cell lineages while existing in the periphery. The variability of monocytes is suitable for maintaining homeostasis flexibly according to the situation. In this study, we found that apoptosis and functional alterations are induced in monocytes early after systemic inflammation, which effectively suppresses the appearance of monocytes in peripheral tissues. During excessive inflammation, this could be a mechanism to attenuate monocyte-mediated acceleration of systemic inflammation and tissue damage and instead assure host survival. Indeed, suppression of monocyte appearance was observed during severe inflammation both in mice and in humans.

Apoptosis maintains homeostasis by silently removing unwanted or harmful cells during various biological processes such as development, inflammation, and aging (36–38). Importantly, the impaired apoptotic cell death selectively in monocytes, i.e., *Casp3/7^Δmono^* mice, exacerbated the inflammation and increased host death due to LPS-induced sepsis (**Figure 5**), which suggests that the apoptosis-mediated reduction of monocytes is a novel fine-tuning mechanism to prevent excessive inflammation. In this respect, non-apoptotic cell death such as necrosis was also suggested to increase inflammation and decrease host survival. Further research will be needed to elucidate whether this tuning machinery is active in various diseases and whether its impairment leads to the onset of inflammatory diseases. In addition, the functionally altered monocytes had a decreased ability to produce pro-inflammatory cytokines, such as TNF-α and CXCL1 (**Figure 3H**). In contrast, their ability to produce anti-inflammatory cytokines was not upregulated (**Figure 3H**), suggesting that the dysfunctional monocytes seem not to be suppressive monocyte subsets such as Ym1^+^ monocytes (14). Interestingly, monocytes that survived in the BM after LPS treatment showed a remarkable expression of G-CSF (**Figure 3H**). As G-CSF induces the differentiation of neutrophils and Ym1^+^ monocytes (39, 40), the functional alteration of monocytes may affect subsequent immune responses at the later phase of inflammation. In addition, the reduced production of TNF-α and the enhanced production of G-CSF are observed in monocytes of human septic patients (41), implying that a similar functional alteration of monocytes during inflammation is widely conserved in mammals. Our findings indicated that inflammatory cytokines induce the apoptosis and functional alterations of monocyte in the periphery. With this respect, the functional alternations of monocytes, such as reduced TNF-α production, might be caused by hypoxia- or LPS-mediated upregulation of hypoxia inducible factor-1α (HIF1α) that reduces inflammatory cytokine production from monocytes (42, 43).

The results of this study show that “monocyte loss” is triggered in the periphery only under severe inflammation (**Figures 7F, G**, **4E, F**; **Supplementary Figures 2F**, **5**), which was transient and canceled as soon as the inflammation subsided both in mice and in humans (**Figures 1A**, **7B**). On the other hand, it has been considered that a dysregulated immune response is induced in septic patients, e.g. the induction of indiscriminate apoptosis in a variety of cell types. However, we found that the susceptibility of monocytes to apoptosis is higher than that of other immune cells (**Figures 2D, E, H, I**; **Figure 6L**), which leads to the preferential removal of monocytes in a programmed manner. In contrast to the monocyte loss under excessive inflammation, monocyte tolerance is induced during mild inflammation triggered by low doses of LPS (44–46). Mechanistically, the monocyte loss was induced by pro-inflammatory cytokines independent of microbial stimuli (**Figures 6G–L**). Consistently, monocyte loss was also observed in patients with CAR-T cell-induced CRS (**Figure 7**), implying that monocyte loss is a common regulatory mechanism that limits excessive inflammation both in mice and in humans.

In another aspect, previous studies on monocyte- or monocyte-derived cell-mediated immune suppression have focused primarily on the immunological significance during the late stage of inflammation, such as sepsis, inflammatory bowel disease, and allergy (2, 14, 47, 48). Depending on the immunological context, the late stage of monocyte suppression may increase patient mortality caused by secondary infections (2, 49, 50), or it may help to terminate inflammation and promote tissue repair (14, 42, 46, 47). In contrast to these studies, we focused on the early inhibitory mechanism of hyperinflammation and newly identified that the transient reduction and dysfunction of monocytes fine-tunes the magnitude of inflammation to minimize tissue damage and to assure host survival.

The recruitment of monocytes to infected sites is often later than that of neutrophils (46, 51). We found that the more severe the inflammation, the more delayed the appearance of monocytes in the periphery, which may explain the reason for the delayed monocyte recruitment to the infected site. While previous studies with low-dose LPS treatment showed a mobilization of monocytes from the BM to peripheral tissues (26), the monocyte behavior in severe inflammatory conditions has not been fully elucidated. Supporting our conclusion, Giamarellos-Bourboulis et al. reported that septic patients with apoptosis in more than 50% of their peripheral blood monocytes have a lower mortality compared to patients with apoptosis in less than 50% of their monocytes (52). Although their study did not prove the mechanism and causal relationship involved, it implies the apoptosis-mediated removal of monocytes with the suppression of systemic inflammation.

In this study, we used blood samples from CRS patients to elucidate whether monocyte disappearance is induced in systemic inflammatory conditions. Because CRS is triggered after CAR-T cell therapy, it is easy to monitor the progress of inflammation and cell kinetics from before the onset of inflammation. The tumor burden before CAR-T cell injection and CAR-T cell expansion is a risk factor for CRS development in cancer treatment (53, 54). However, because accurate evaluation of the tumor burden requires analysis with computed tomography (CT), magnetic resonance imaging (MRI), and/or positron emission tomography/CT (PET/CT) scanning, the identification of biomarkers that can easily predict the severity of CRS would be beneficial. Here, we demonstrate that the number of classical monocytes in the patient’s blood before CAR-T cell administration is a useful biomarker to predict the severity of CRS. The high number of classical monocytes in the periphery before CAR-T cell administration correlated with the severity of CRS, implying that monocytes are a major source of pro-inflammatory cytokines. Supporting this notion, the importance of monocytes as a source of inflammatory cytokines during CRS induced by CAR-T cell therapy was indicated in a humanized mouse model (35). Meanwhile, as the development of CRS does not correlate with the prognosis of primary disease (55), our findings further suggested that the classical monocyte counts cannot predict the outcomes of CAR-T cell therapy. In addition, temporary removal of monocytes may help control systemic inflammation, although prolonged monocyte depletion risks increased susceptibility to infection and collapse of homeostasis. Of note, we previously developed the antibody-drug conjugate (ADC) that selectively removes human monocytes and monocytic leukemia cells with minimal side effects on other hematopoietic cells by targeting monocyte progenitors (56). This ADC might also be applicable for CRS, although this is an issue for the future.

To avoid uncontrolled inflammation, the immune system is equipped with various regulatory mechanisms that function appropriately at the right time. In this study, we found the apoptotic removal and induction of dysfunctionality in monocytes at early stages of excessive inflammation (**Supplementary Figure 5**). Because this mechanism does not need help with adaptive immunity, it might be a primitive system that has existed since before the development of the acquired immune system. Considering this novel fine-tuning mechanism, our findings are valuable not only for extending our understanding of monocyte function but also for identifying therapeutic targets for severe inflammatory diseases.

## Data availability statement

The original contributions presented in the study are included in the article/**Supplementary Material**. Further inquiries can be directed to the corresponding author.

## Ethics statement

The studies involving humans were approved by the Institutional Review Board of the Tokyo Medical and Dental University. The studies were conducted in accordance with the local legislation and institutional requirements. The participants provided their written informed consent to participate in this study. The animal study was approved by the Institutional Animal Care Committee of the Tokyo Medical and Dental University. The study was conducted in accordance with the local legislation and institutional requirements.

## Author contributions

TO: Conceptualization, Data curation, Project administration, Supervision, Validation, Writing – original draft, Writing – review & editing. MA: Data curation, Formal analysis, Investigation, Writing – review & editing. MK: Conceptualization, Data curation, Formal analysis, Funding acquisition, Investigation, Methodology, Validation, Writing – original draft, Writing – review & editing. YU: Investigation, Methodology, Resources, Writing – review & editing. TN: Investigation, Methodology, Resources, Writing – review & editing. YI: Formal analysis, Investigation, Methodology, Writing – review & editing. MY: Investigation, Methodology, Resources, Writing – review & editing.
