# Supplementary material for: An early regulatory mechanism of hyperinflammation by restricting monocyte contribution
Source: Front Immunol. 2024 Jul 8;15:1398153. doi: 10.3389/fimmu.2024.1398153 (PMC11260625; doi:10.3389/fimmu.2024.1398153)
Supplement: Supplementary file 1 [file DataSheet_1.pdf]

Supplementary Figure 1

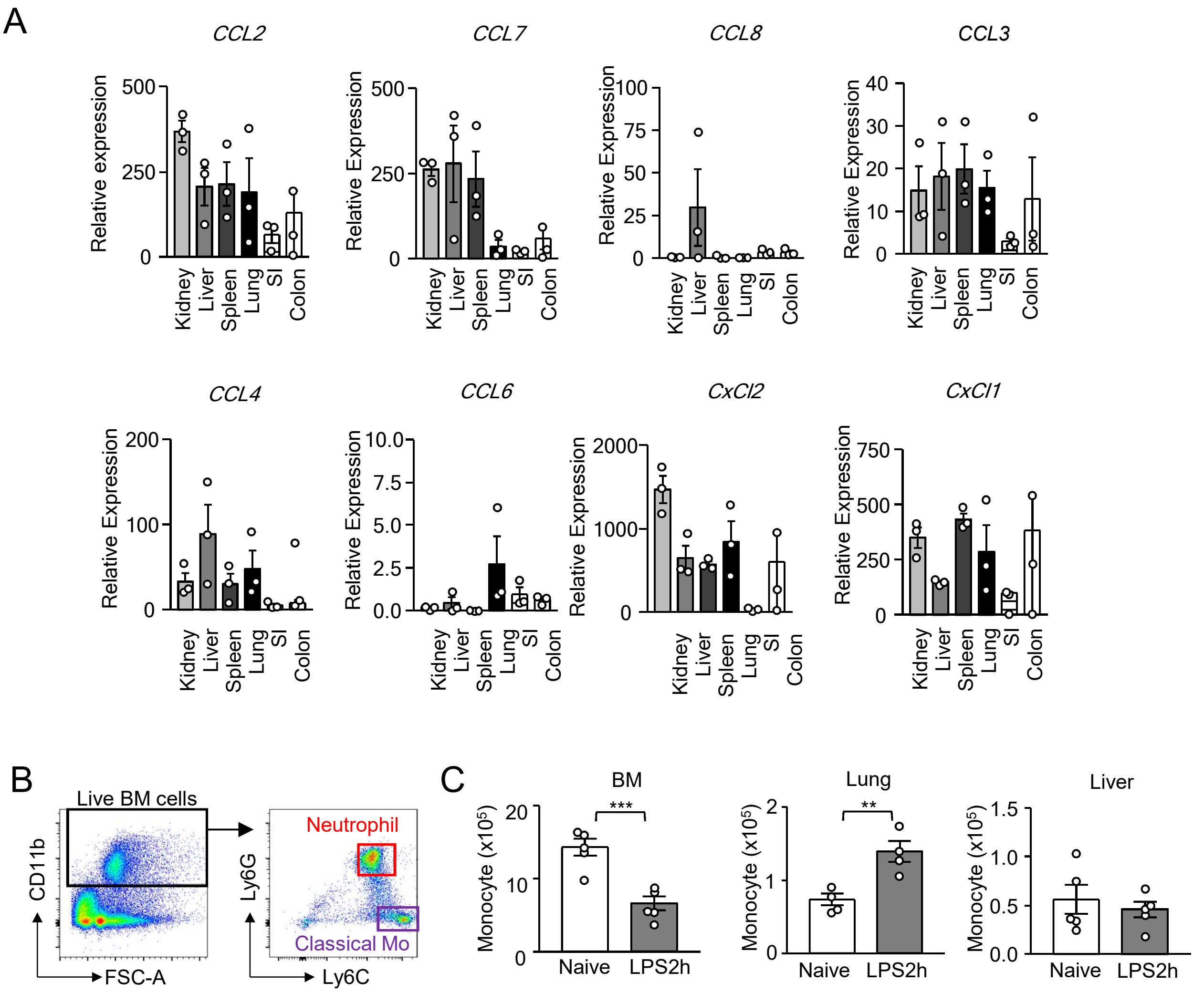

Supplementary Figure 1

Upregulation of chemokines for monocytes and neutrophils after LPS treatment. (**A**) Tissues were harvested 5 hours after treatment with LPS (5 mg/kg) and mRNAs were isolated. Gene expression levels of the indicated chemokines were examined using qPCR; data are shown as fold change of gene expression levels before and after treatment. (**B**) Gating strategies for flow cytometry; classical monocytes and neutrophils were identified as $CD11b^{+}Ly6G^{-}Ly6C^{hi}$ and $CD11b^{+}Ly6G^{+}Ly6C^{-}$ cells, respectively. PI^{-} -singlet cells were pre-gated before gating with markers. (**C**) Number of monocytes in the BM, lung and liver 2 hours after LPS-treatment (5 mg/kg). **p<0.01, ** p<0.001, N.S.; not significantly different. (Student's t-test [C]). Data are pooled from two independent experiments (C) (error bars, SEM).

Supplementary Figure 2

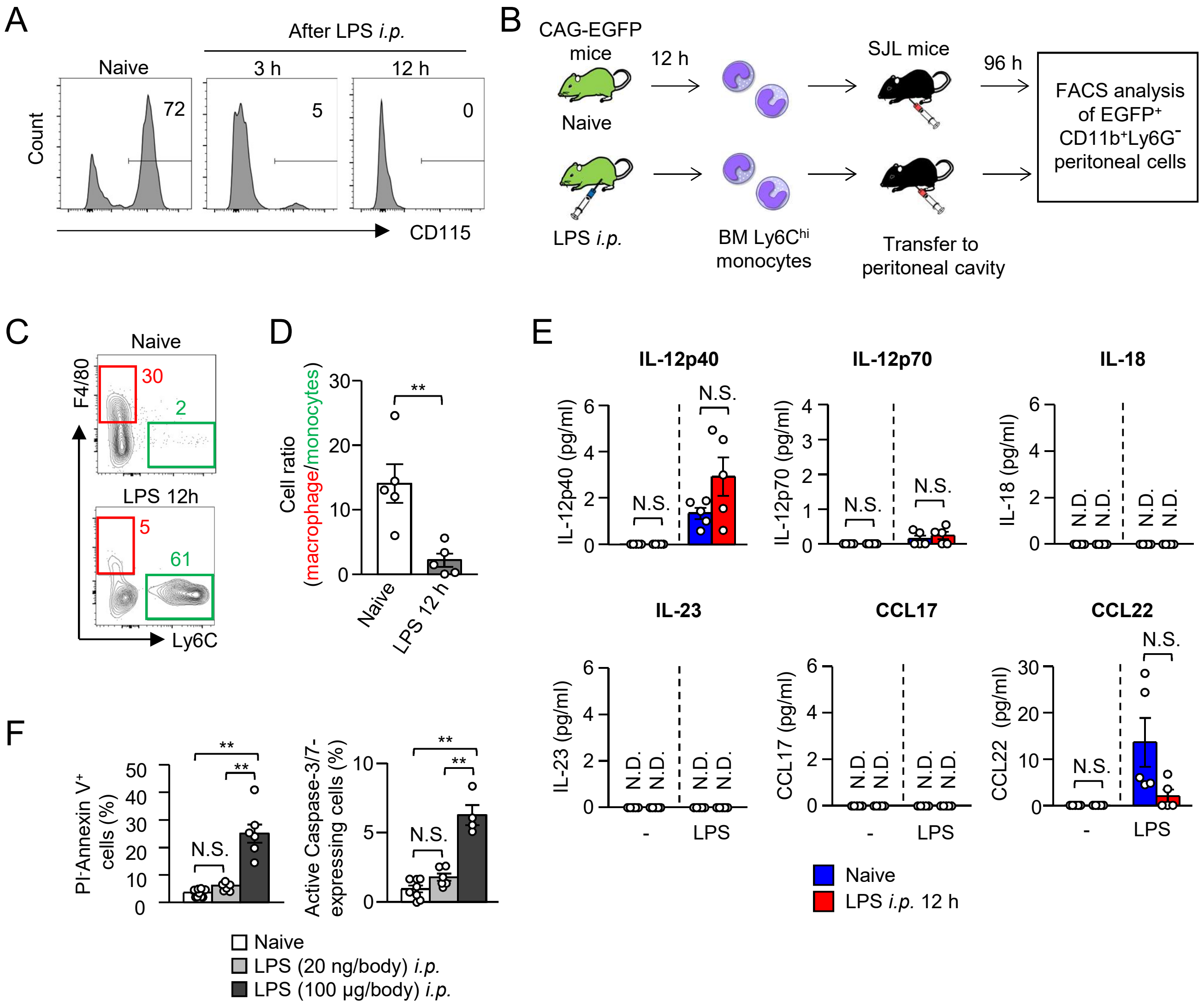

Supplementary Figure 2

Functional defects in BM monocytes after LPS treatment. **(A)** Expression of CD115 on the surface of monocytes in the BM before and 3 and 12 hours after LPS treatment (5 mg/kg). **(B-D)** Ex vivo macrophage-differentiation assay of BM monocytes; monocytes obtained from the BM of naïve or LPS-treated CAG-EGFP mice were transferred into the peritoneal cavity of naïve SJL mice. Four days after the transfer, the frequencies of monocytes and macrophages were examined using flow cytometry. The experimental strategy is shown in (B). Representative FACS plots and ratios between macrophage and monocytes are shown in (C) and (D), respectively; n=3 per group. The numbers on the FCM plots indicate the frequencies of the gated populations. **(E)** Cytokine production by monocytes obtained from the BM of WT mice before and 12 hours after LPS treatment (5 mg/kg) cultured overnight in the presence of LPS (100 ng/ml) after which the supernatants were collected for analysis with Legendplex. **(F)** The ratio of early apoptotic cells (PI^- Annexin V^+) and dead cells (PI^+ Annexin V^+) in BM monocytes 12 hours after treatment with the indicated amount of LPS. *p< 0.05, **p<0.01, N.S.; not significantly different, N.D.; not detectable (Student's t-test [D, E] and one-way ANOVA [F]). Data are representative of two independent experiments (A) or pooled from two independent experiments (B-F) (error bars, SEM).

Supplementary Figure 3

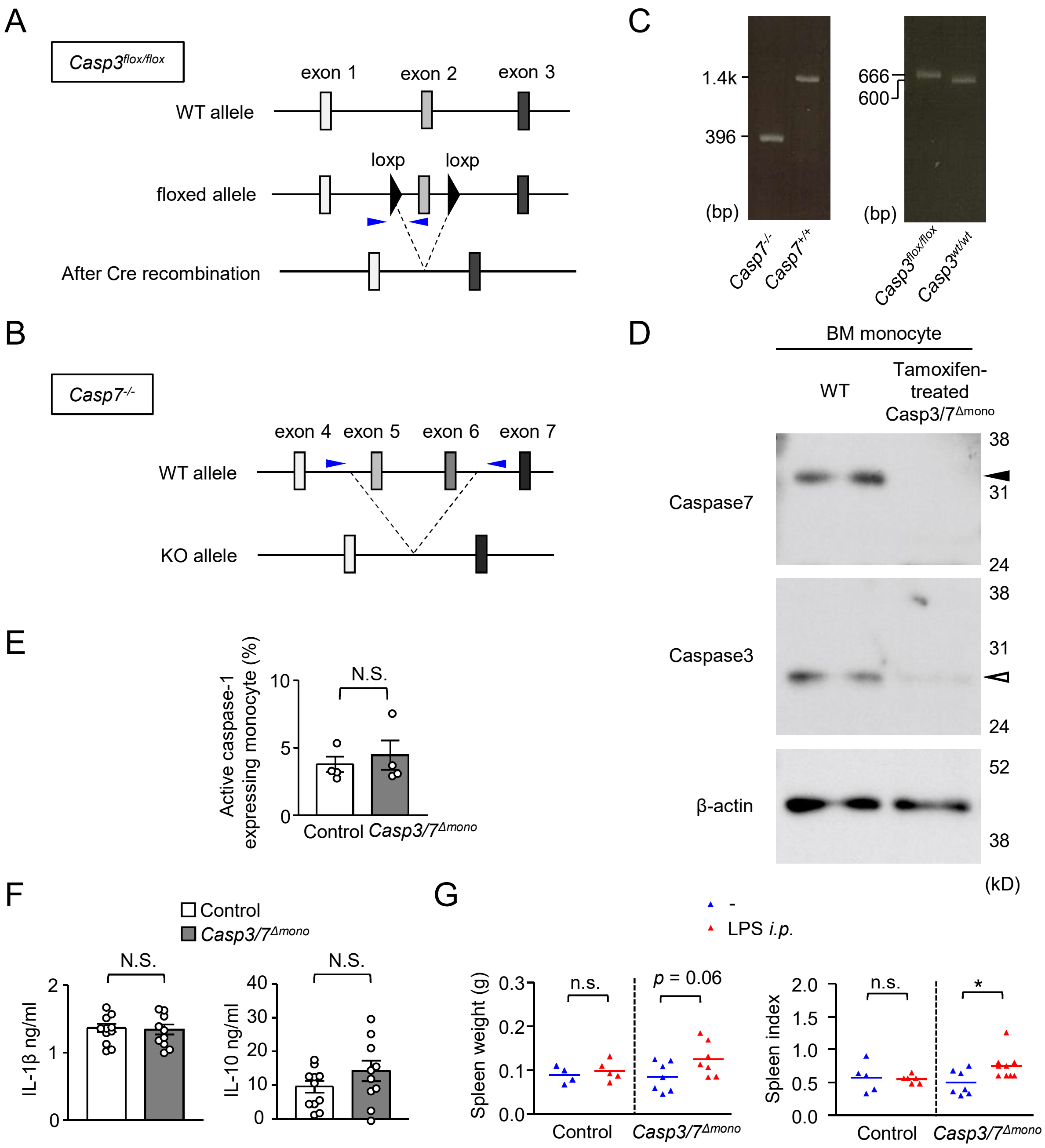

Supplementary Figure 3

Generation of *Casp3$^{flox/flox}$* mice and *Casp7$^{-/-}$* mice. (**A, B**) Constructs for *Casp3-flox* and *Casp7* knock-out; boxes indicate exons of *Casp3* or *Casp7*. (**C**) Genotyping for *Casp3-flox* and *Casp7$^{-/-}$*. Insertion of the loxp sequence in the *Casp3*-floxed allele and the deletion of *Casp7* exons 5 and 6 were confirmed by PCR using specific primers (blue arrowheads in A and B). (**D**) Relative quantification of Caspase-3 and Caspase-7 in BM monocytes obtained from Casp3/7$^{\Delta mono}$ mice after 5 tamoxifen injections assessed by western blotting; BM monocytes in WT mice were used as a control. Black and white arrowheads indicate bands of Caspase-3 and Caspase-7, respectively. (**E**) Frequency of active caspase-1^{+} cells in BM monocytes 13 hours after LPS treatment (5 mg/kg). (**F**) Plasma cytokine levels 2 hours after LPS treatment (5 mg/kg) of control mice or *Casp3/7$^{\Delta mono}$* mice; n=10 per group. (**G**) Spleen weight and index of control and *Casp3/7$^{\Delta mon}$* mice; the spleen index was calculated by multiplying the short axis and long axis 12 hours after LPS treatment (5 mg/kg); n=7 for each group in (F). *p<0.05, N.S.; not significantly different (Student's t-test). Error bars, SEM. Data are representative of two independent experiments (C, D, E) or pooled from two independent (F, G) experiments.

Supplementary Figure 4

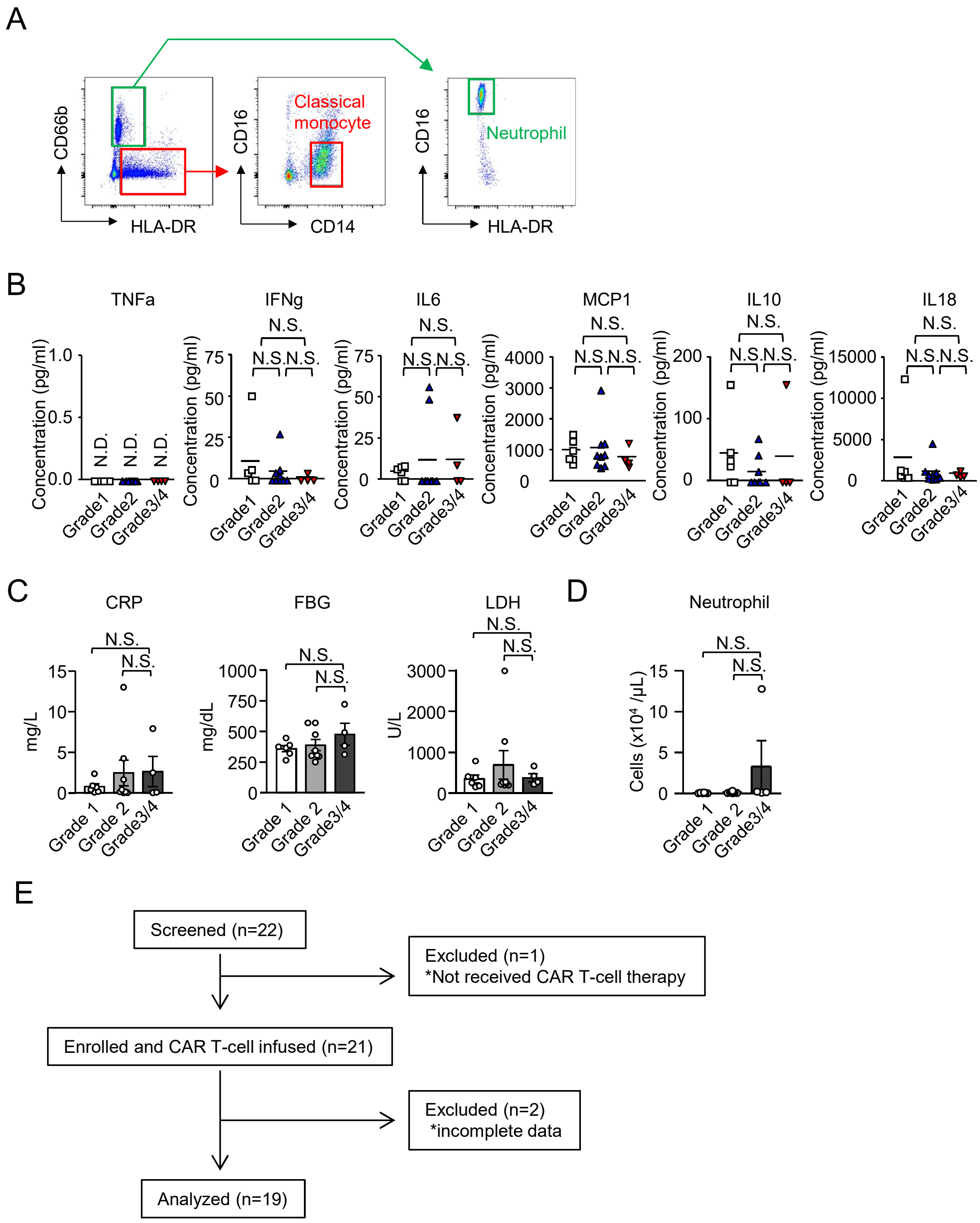

Supplementary Figure 4

The severity of CRS can not be anticipated by the levels of inflammation markers, blood neutrophil number, or total monocyte number at day 0. (**A**) Gating strategy of human classical monocytes and neutrophils. (**B-D**) Levels of cytokines (B), inflammation markers (CRP, FBG, and LDH) (C), and neutrophil numbers (D) in the peripheral blood obtained from CRS patients immediately before CAR-T cell injection (day 0). (**E**) Consort diagram of CAR-T cell therapy study. *** p<0.001, N.S.; not significantly different (Student's t-test). Error bars, SEM.

Supplementary Figure 5

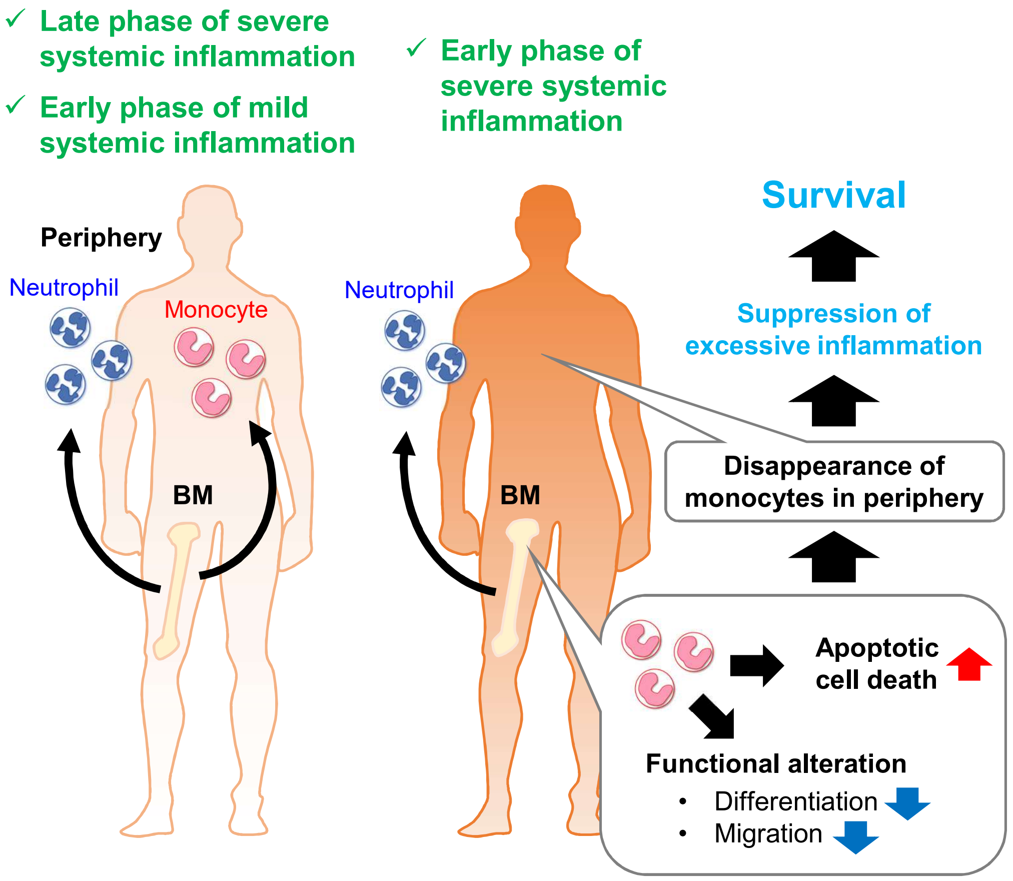

Supplementary Figure 5. Graphical summary of this study.

Supplementary Table 1. CRS grading scale

Grade	Penn scale
Grade 1	• Mild reaction treated with supportive care only
Grade 2	• Moderate reaction requiring IV therapies or parenteral nutrition<br>• Mild signs of organ dysfunction (creatinine ≤ grade2 or 4 liver function test results ≤3)<br>• Hospitalization for CRS or febrile neutropenia
Grade 3	• More severe reaction requiring hospitalization<br>• Moderate signs of organ dysfunction (grade 3 creatinine or grade 4 liver function test results) related to CRS<br>• Hypotension treated with IV fluids or low-dose pressors<br>• Coagulopathy requiring FFP or cryoprecipitate<br>• Hypoxemia requiring oxygenation (nasal cannula oxygen, high flow O2, BiPAP, CPAP)
Grade 4	• Life-threatening complications, including hypotension requiring high-dose vasopressors or hypoxemia requiring mechanical ventilation
Grade 5	• Death

BiPAP, biphasic positive air pressure CPAP, continuous positive air pressure

Supplementary Table 2. Patient characteristics of CAR-T cell therapy.

	UPENN CRS grade	1	2	3	4
Total, n (%) n=19		6(31.6)	9(47.4)	2(10.5)	2(10.5)
Sex, n	Female/Male	3/3	3/6	0/2	0/2
Median age at CAR-T cell therapy (range), y		58.5(53-74)	58(25-72)	62.5(50-69)	62.5(46-79)
Disease, n	B-ALL	0	1	0	0
	DLBCL	6	8	2	2
Prior ASCT, n (%)		2(33)	2(25)	1(50)	0
Disease status, n	CR/PR/PD/SD	0/3/2/1	2/3/2/0	0/2/0/0	0/1/1/0
Dose reduction of conditioning regimen, n (%)		0	1(11)	0	2(100)
ICANS, n (%)		0	1(11)	0	1(50)
Any corticosteroid treatment, n (%)		2(33)	3(33)	2(100)	2(100)
tocilizumab treatment, n (%)		2(33)	8(89)	2(100)	2(100)
G-CSF treatment after infusion, n (%)		1(17)	1(14)	0	1(50)
Infection, n	Bacterial/ Viral	0/1	0/1	0/0	0/0

Abbreviations:
B-ALL: B-cell acute lymphoid leukemia, DLBCL: diffuse large B-cell lymphoma
ASCT: autologous stem cell transplantation
CR: complete remission, PR: partial response, PD: progressive disease, SD: stable disease
ICANS: immune effector cell- associated neurotoxicity syndrome

Supplementary Table 3. Cytokine profiles of CAR T-cell therapy at Day0.

Patient #	IL-1β (pg/ml)	IFN-α2 (pg/ml)	IFN-γ (pg/ml)	TNF-α (pg/ml)	MCP-1 (pg/ml)	IL-6 (pg/ml)	IL-8 (pg/ml)	IL-10 (pg/ml)	IL-12p70 (pg/ml)	IL-17A (pg/ml)	IL-18 (pg/ml)	IL-23 (pg/ml)	IL-33 (pg/ml)
#1	0	0	2.822	0	873.69	0	0	0	0	0	923.5	0	0
#2	0	0	0	0	530.6	0	0	0	0	0	418.2	0	0
#4	0	0	0	0	889.41	0	0	0	0	0	400.57	0	0
#6	0	0	4.508	0	1250.01	0	0	17.06	0	0	1211.13	0	0
#7	0	0.89	27.897	0	2975.45	56.9	0	70.13	0	0	4690.87	0	0
#8	0	2.22	6.183	0	1227.35	49.46	0	43.2	0	0	1337.7	26.11	25.1
#9	14.86	1.88	4.508	0	768.53	8.98	0	34.57	8.96	0	1330.79	31.97	51.3
#10	0	1.75	2.822	0	583.58	5.33	0	48.17	8.25	0	575.41	0	332.9
#11	0	0	0	0	499.85	9.52	0	0	0	0	647.29	0	0
#12	0	0	0	0	1028.67	6.98	0	0	0	0	1476.23	0	0
#13	0	1.1	51.114	0	1552.13	8.3	0	157.4	0	0	12546.61	0	0
#14	0	0	0	0	573.95	0	0	0	0	0	491.61	0	0
#15	0	0	0	0	1327.74	0	0	0	0	0	857.52	0	0
#16	0	0	6.34	0	747	0	0	25.75	0	0	605.38	0	0
#17	0	0	0	0	721.71	0	0	0	0	0	913.29	0	0
#18	0	0	4.215	0	1265.03	38.37	0	157.81	0	0	1409.99	0	0
#20	0	0	0	0	472.87	0	0	0	0	0	922.15	0	0
#21	0	0	0	0	630.29	0	0	0	0	0	812.09	0	0
#22	0	0	0	0	836.8	0	0	0	0	0	577.51	0	0

Supplementary Table 4. Primer sequences for qPCR analysis.

Gene	Forward Primer	Reverse Primer
Ccl2	ggcctgctgttcacagtgc	cctgctgctggtgatcctctt
Ccl3	actgcctgctgcttctcctacaa	aggaaaatgacacctggctgg
Ccl4	aaacctaaccccgagcaaca	ccattggtgctgagaaccct
Ccl6	aagaagatcgtcgctataaccct	gcttaggcacctctgaactctc
Ccl7	aagatccccaagaggaatctcaag	cagacttccatgcccttctttg
Ccl8	cgcagtgcttctttgcctg	tctggcccagtcagcttctc
CxCl1	tgggattcacctcaagaaca	tttctgaaccaagggagctt
CxCl2	ccaccaaccaccaggctac	gcttcagggtcaaggcaaa
Actb	tgttaccaactgggagcgaca	ctgggtcatcttttcacggt
